# The BE-UJI hand function activity set: a reduced set of activities for the evaluation of the healthy and pathological hand

**DOI:** 10.1186/s12984-023-01245-1

**Published:** 2023-09-21

**Authors:** Néstor J. Jarque-Bou, Verónica Gracia-Ibáñez, Margarita Vergara, Joaquín L. Sancho-Bru

**Affiliations:** Biomechanics and Ergonomics Group, Department of Mechanical Engineering and Construction, Universitat Jaume I, Avinguda Vicent Sos Baynat, s/n., 12071 Castellón, Spain

**Keywords:** Activities of daily living, Hand osteoarthritis, Hand synergies, Hand function, Principal component analysis, Kinematics, Rehabilitation, Sollerman Hand Function Test

## Abstract

**Background:**

Hand kinematics during hand function tests based on the performance of activities of daily living (ADLs) can provide objective data to determine patients’ functional loss. However, they are rarely used during clinical assessments because of their long duration. Starting with the 20 Sollerman Hand Function Test (SHFT) tasks, we propose identifying a reduced set of ADLs that provides similar kinematic information to the original full set in terms of synergies, ranges of motion and velocities.

**Methods:**

We followed an iterative method with the kinematics of 16 hand joints while performing the 20 ADLs of the SHFT. For each subject, ADLs were ordered according to their influence on the synergies obtained by means of a principal component analysis, the minimum number of ADLs that represented the original kinematic synergies (maximum angle of 30° between synergies), and the maintained ranges of joint movements (85% of the original ones) were selected for each subject. The set of the most frequently selected ADLs was verified to be representative of the SHFT ADLs in terms of motion strategies, ranges of motion and joint velocities when considering healthy subjects and Hand Osteoarthritis patients.

**Results:**

A set of 10 tasks, the BE-UJI activity set, was identified by ensuring a certain (minimum) similarity in synergy (maximum mean angle between synergies of 25.5°), functional joint ranges (maximum differences of 10°) and joint velocities (maximum differences of 15°/s). The obtained tasks were: pick up coins from purses, lift wooden cubes, pick up nuts and turn them, write with a pen, cut with a knife, lift a telephone, unscrew jar lids and pour water from a cup, a jar and a Pure-Pak. These activities guarantee using the seven commonest handgrips in ADLs.

**Conclusion:**

The BE-UJI activity set for the hand function assessment can be used to obtain quantitative data in clinics as an alternative to the SHFT. It reduces the test time and allows clinicians to obtain objective kinematic data of the motor strategies, ranges of motion and joint velocities used by patients.

**Supplementary Information:**

The online version contains supplementary material available at 10.1186/s12984-023-01245-1.

## Background

The human hand is a sophisticated biological and mechanical system that confers humans the manipulative capacity to interact with their physical environment and to perform activities of daily living (ADLs). The hand function may be defined as the capacity to use our hands in ADLs depending on anatomical coordination, strength and dexterity [1]. Different pathologies and injuries can affect the hand function, and range from accidents and occupational pathologies to neurological diseases, which diminish the ability to perform ADLs. Assessing hand function is key for determining the extent of patients’ functional loss [1]. The World Health Organization [2] establishes that this assessment should be based on the objective evaluation of the capabilities to perform ADLs. Currently, the structural and functional impairment of hands are measured mainly by passive ranges of motion with goniometry [3–6], force measurements using dynamometers [7], sensory assessment [8] and functional tests [9, 10] (e.g. nine-hole peg test or functional dexterity test). Range measurement has the added problem of being very dependent on operator intent insofar as the passive range values obtained by an orthopaedic surgeon, rehabilitator or disability assessor may differ [8]. During the rehabilitation process or clinical planning, the objective range measurements are, in any case, combined with highly subjective tests or specific scales for each pathology [9–11]. However, performing ADLs is rarely assessed in current clinical practice, and is at best limited to questionnaires completed by patients on their ADL skills [12].

Some previous works have reviewed the tests and scales used in clinical practice or in rehabilitation [13–15]. Metcalf et al. [13] identified 25 different clinical upper limb assessment methods within the ICF framework. Medical professionals must choose the most appropriate method to evaluate a specific pathology based on studies about the sensitivity and validity of the method for that pathology. Of all these methods, very few tests for the hand function assessment based on ADLs exist, such as the Jebsen hand function test [16] and the Sollerman Hand Function Test (SHFT) [17]. The latter considers more varied and representative ADLs, which have been selected based on the commonest hand grasps [17]. However, the only objective parameter measured in these tests is the time taken to accomplish tasks, together with the used grasp type, which is identified by the operator with visual observation. Monitoring hand kinematics while performing these tests has been recently proposed as a way to obtain more accurate and objective information about motor strategies, which also leads to a better application of therapeutic strategies [18].

However, some major obstacles that hinder the monitoring of hand kinematics during these tests in clinical practice are: (1) the difficulty of analysing the large number of degrees of freedom (DoF) used simultaneously; (2) the long duration of tests because many activities have to be recorded. This latter issue is critical in clinics because of either evaluation times or the discomfort that people with pathologies may suffer during long tedious testing.

Recently, the first obstacle has already been approached by means of kinematic synergies [19]. Although hand motion has many DoF, not all the joint movements are independent due to mechanical and neural couplings. The coordinated movements between various joints resulting from these couplings are referred to as kinematic synergies. Therefore, kinematic synergies are suggested as a method to represent the basic building blocks that underlie natural hand motions, and can be used to reduce the dimensionality of hand kinematics. The principal component analysis (PCA) is the most widely used method for dimensionality reduction. This method looks for linear combinations of correlated variables to find a reduced set of new uncorrelated variables. In previous studies of the authors [18, 20], kinematic synergies have been applied to reconstruct the full hand kinematics by recording only a few joint angles and estimating the remaining angles from the coordination established by those synergies. This has been verified to be feasible in not only healthy subjects [20], but also in patients [18].

Therefore, the aim of this work is to approach the second obstacle. To do so, a set of activities for the hand function assessment with a few ADLs is proposed, hereafter referred to as the BE-UJI (Biomechanics and Ergonomics group of the Universitat Jaume I) activity set. Furthermore, the BE-UJI activity set has been tested on an orthopaedic disability, such as Hand Osteoarthritis (HOA), although it is not limited to or focuses on any type of hand disability. A test with a selection of a few, but sufficient activities to monitor hand kinematics would increase efficacy and reduce the time and cost of current rehabilitation and assessment programmes, while allowing more objective and accurate parameters of motor strategies to be obtained. The SHFT is a widely used and validated test that evaluates hand function [9, 21–24] and is based on the commonest hand grasps used in ADLs [17]. Furthermore, the SHFT has recently been used to compare the hand kinematics of HOA patients with the kinematics of the healthy hand, and significant differences in both the SHFT score and ranges of motion have been observed [25]. Therefore, by starting with the SHFT ADLs, we followed an iterative method to reduce the number of ADLs while keeping the kinematics required for functionality in representative ADLs in terms of motion strategies, ranges of motion and velocities. Finally, the BE-UJI activity set was evaluated in these terms in two different populations: healthy subjects and patients with HOA.

## Methods

### Experimental study

**Fig. 1:**
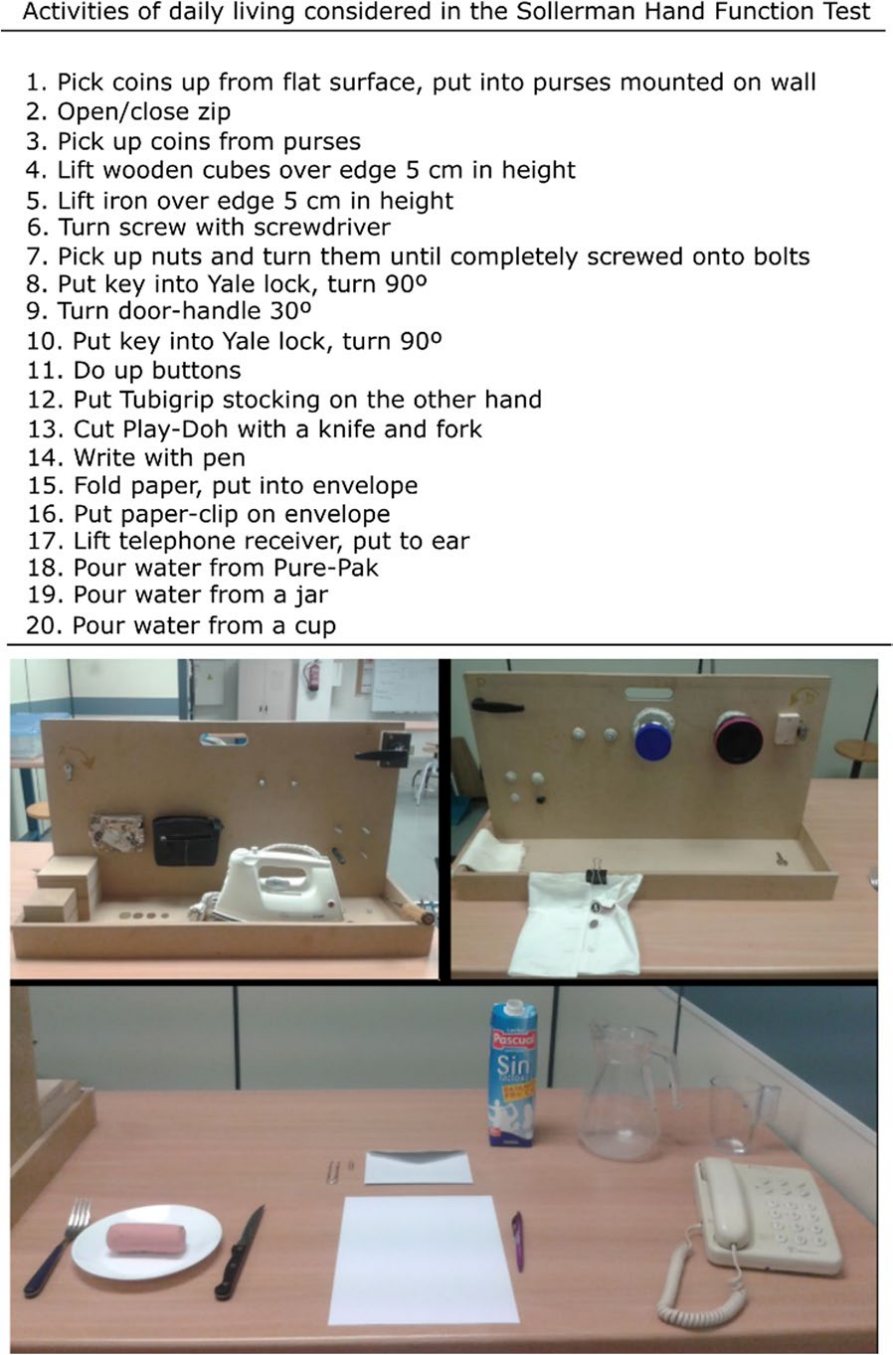
Activities and scenarios of the Sollerman Hand Function Test

The hand kinematics data of 27 healthy subjects (hereafter referred to as the healthy cohort) while performing the 20 SHFT tasks were employed [25] (Fig. 1). Each subject performed the 20 ADLs under controlled laboratory conditions using real objects and following the original test instructions [17]. The healthy cohort was recruited from among research team members, staff of the university and their relatives, and students. The inclusion criteria considered subjects without a history of neuromuscular problems or injuries of the upper arm. All the participants provided their informed consent to participate in the experiment (approved by the Research Ethics Committee with Human Beings, CD/31/2019).

**Fig. 2:**
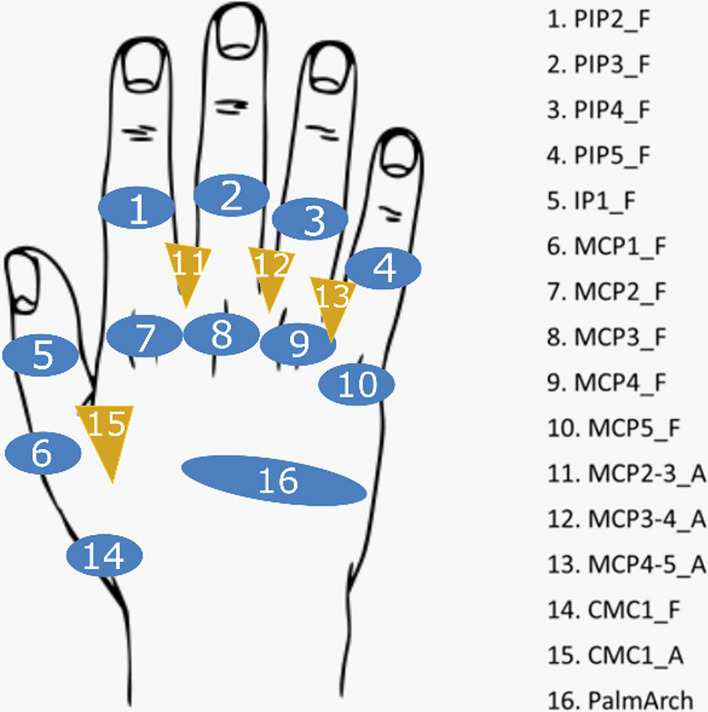
Joint angles recorded. Nomenclature: _F for flexion (in blue), _A for abduction of thumb and relative abduction between fingers (in yellow); 1 to 5, digits. Joints: IP for interphalangeal joint, PIP for proximal interphalangeal joints, MCP for metacarpophalangeal joints, CMC for carpometacarpal joints, PalmArch for palmar arch

Sixteen joint angles were recorded (Fig. 2) at 100 Hz with an instrumented glove [Cyberglove Systems LLC; San Jose, CA (USA)] following a validated calibration protocol [26]: flexion of metacarpophalangeal joints (MCP1 to MCP5, 1 to 5 meaning thumb to little digits), flexion of interphalangeal thumb joint (IP1), flexion of the proximal interphalangeal joints of fingers (PIP2 to PIP5), flexion and abduction of the carpometacarpal thumb joint (CMC1), relative abduction between the finger MCPs (index-middle, middle-ring, and ring-little) and palmar arching. The positive values of angles correspond to flexion and abduction. Recordings were filtered with a 2nd-order 2-way low-pass Butterworth filter at a cut-off frequency of 5 Hz.

A synergy-based methodology, presented below, was used to find the minimum number of ADLs that provided similar kinematic information to that obtained by considering all the SHFT ADLs in terms of the most relevant kinematic parameters.

### Analysis outline

- Step 1. Extracting the subject-specific kinematic synergies that underlie the 20 SHFT ADLs through the computation of PCs. The resulting set of synergies (Ref PCs) was considered a reference for the next steps.
- Step 2. For each subject, ordering ADLs from least to greatest effect on the Ref PCs if removed. In each step, the subtracted ADL was that with the least effect on the resulting synergies when removed in comparison to the Ref PCs. This process was repeated until only one ADL remained, and the result was a vector of ADLs sorted according to the order of subtraction.
- Step 3. For each subject, selecting the minimum number of ADLs, from the sorted ADLs of the previous step, which provided a range of movement of at least 85% of that of the original data, and kept joint coordination in terms of synergies (maximum angle between k-PCs and the Ref PCs equalled or was lower than 30°).
- Step 4. Selecting the reduced set of ADLs for the hand function assessment as those that appeared more frequently in all the subjects.
- Step 5. Verifying that the reduced set of ADLs was the equivalent to the original set of ADLs in terms of motion strategies, ranges of motion and velocities of hand joints in two different populations; the healthy cohort and patients with HOA.

**Fig. 3:**
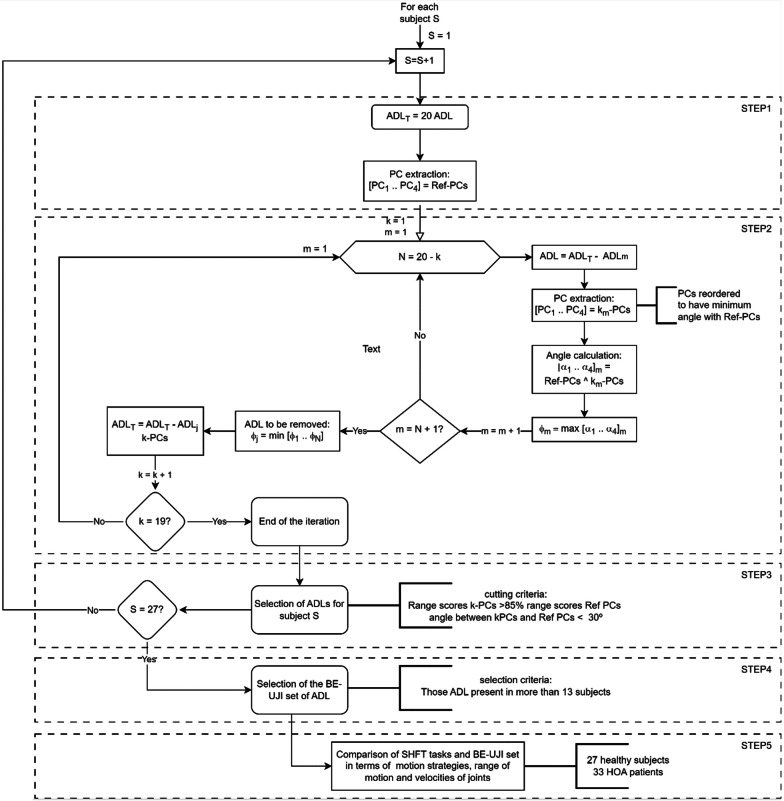
Scheme of the method applied per subject

Table 1 presents the details of the key items involved in each step, which are better detailed in “Detailed analysis” section. Figure 3 outlines the entire followed process.

**Table 1:** Key items and results obtained in each step followed in the methodology

Step	Description	Details
1	Extracting kinematic PCs	Results Set of specific-subject synergies used as a reference in the following steps (Ref-PCs)
2	Ordering ADLs from the least to the greatest effect on Ref-PCs	Results Vector of ADLs sorted per subject Set of synergies for each iteration k (k-PCs) and per subject
3	Selecting the minimum number of ADLs per subject	Cutting criteria Range scores k-PCs vs. Ref-PCs > 85% Angles k-PCs vs. Ref PCs < 30° Results Minimum number of ADLs per subject
4	Selecting the smallest set of ADLs for h and function assessment	Selection criteria Those ADLs presented in at least half the 27 subjects Results BE-UJI set of ADLs
5	Verifying the main kinematic parameters in two different populations: the healthy cohort and patients with HOA	Results Comparison of motion strategies, ranges of motion and velocities of joints between 20 SHFT tasks and the BE-UJI set

### Detailed analysis

#### Step 1—extracting kinematic PCs

The data analysis was completely performed with the custom-developed software in Matlab. First of all, each SHFT task was resampled to 1000 frames so that all the tasks weighed the same when looking for the underlying synergies. Therefore, the data used throughout the paper consisted of 20 records of 1000 frames for each participant, which resulted in a matrix with 16 columns (joint angles) and 20,000 rows (20 ADL × 1000 frames). Four synergies were extracted per subject (Ref PCs) using PCA and following the methodology presented in previous studies, which considers normalised factors and varimax rotation [27–30]. The loadings of the Ref PCs (representing kinematic synergies) and the corresponding scores (representing the dimensionality-reduced kinematic data) were taken as the descriptive data to be maintained in the ADLs subtraction.

#### Step 2—ordering ADLs from the least to the greatest effect on the Ref PCs if removed

An iterative subtraction method was implemented based on the PCA. The PCA was applied per subject to avoid any unrealistic synergies that could appear when merging data from different subjects [28]. The scheme of the used method is shown in Fig. 3 (step 2). For each subject, the data of each ADL were tentatively removed one by one, and the resulting N datasets were used as input in N PCAs (one PCA, also with four extracted PCs, for every removed ADL; N = 20 − k, in the k-th step). Unlike the first kinematic PCA extraction, non-standard scaling was applied [28] using the mean and SD of the original matrix to allow the comparison between the Ref PCs and the PCs from the k-th step (k-PCs). In each k-th step, the angles between the four k-PCs and the Ref PCs vectors were computed to order the k-PCs in terms of similarity with the Ref PCs as in previous works [28]. When determining the similarity between two vectors, the angle formed between them serves as a measure. If vectors are extremely similar or exhibit a strong resemblance, the angle between them will be relatively small. Conversely, if vectors are significantly different or dissimilar, the angle between them will be larger. Thus the angle between two vectors provides insight into their degree of similarity or dissimilarity. In this case, firstly the k-PC with the smallest angle with Ref PC1 (α1), then the k-PC (from the three remaining ones) with the smallest angle with Ref PC2 (α2), and so on. The largest of the angles α1 to α4 was considered an indicator of similarity (α_max) between the original set of the Ref PCs and the new set of the k-PCs. After all the iterations (m = N + 1), the ADL that provided the most similar synergies was removed, i.e., that with the smallest α_max. This iteration was repeated until only one ADL remained (k = 19). For each subject, the vector of the ADLs contained the ADLs ordered from least to greatest effect on the Ref PCs if removed.

#### Step 3—selecting ADLs per subject

For each subject, the cut in the order removal was set at the maximum number of removed ADLs that still met the following criteria: (1) the range (calculated as p95-p5) of the scores of each k-PC should equal or be wider than 85% of the range of the scores of the corresponding Ref PCs; (2) the maximum angle between all four k-PCs and the Ref PCs should equal or be smaller than 30°. The angle of 30° between synergies was set based on the level of similarity between the synergies found in a previous work [28], and the resemblance of 85% in range was set as a high, albeit arbitrary, threshold.

#### Step 4—selecting the reduced set of ADLs for the hand function assessment

From the minimum set of the ADLs of each subject, a selection of the reduced set of ADLs was performed. The selected ADLs were those that were present in more than half the subjects (i.e. in more than 13 subjects) and were those proposed for the BE-UJI set.

#### Step 5—verifying the main kinematic parameters in two different populations: the healthy cohort and patients with HOA

Finally, motion strategies and the main kinematic parameters, such as range of motion and velocities of joints, were compared between the 20 ADLs dataset and the BE-UJI set of ADLs by firstly considering the same dataset used for the ADLs reduction, and by lastly contemplating a dataset previously recorded from a cohort of 33 patients with HOA [25]. HOA patients were recruited by a physical therapist from among hospital patients showing different disease stages and levels of compromise. None had undergone surgery. Samples descriptions, along with significant functional differences, appear in a previous work [25]. For both populations the healthy cohort and HOA patients), the same methodology was followed: firstly for each subject, a PCA was performed by considering the BE-UJI set of ADLs (set PCs). The set PCs were reordered as in step 2 (grouping the more similar set of PCs to the ref PCs), and the motion strategies of each subject were compared by means of the similarity level (angle) between the Ref PCs and the set PCs. Secondly, ranges of motion of joints and velocities were obtained from all the DLs and from the BE-UJI set of ADLs, and were compared by means of box-and-whisker plots.

## Results

### Step 1—composition of the principal components

The explained variance of the four PCs extracted for the subjects was 75.4% ± 2.4%. The first and second synergies were present in almost all the subjects, and mainly involved (Ref PC1) flexion and adduction of fingers MCP joints and (Ref PC2) flexion of PIP joints. The third and fourth synergies were more varied between subjects, but mostly involved two different kinds of coordination: (Ref PC3) coordination between thumb joints; (Ref PC4) coordination between thumb joints and palmar arch.

### Step 2—order of removal of ADLs

**Fig. 4:**
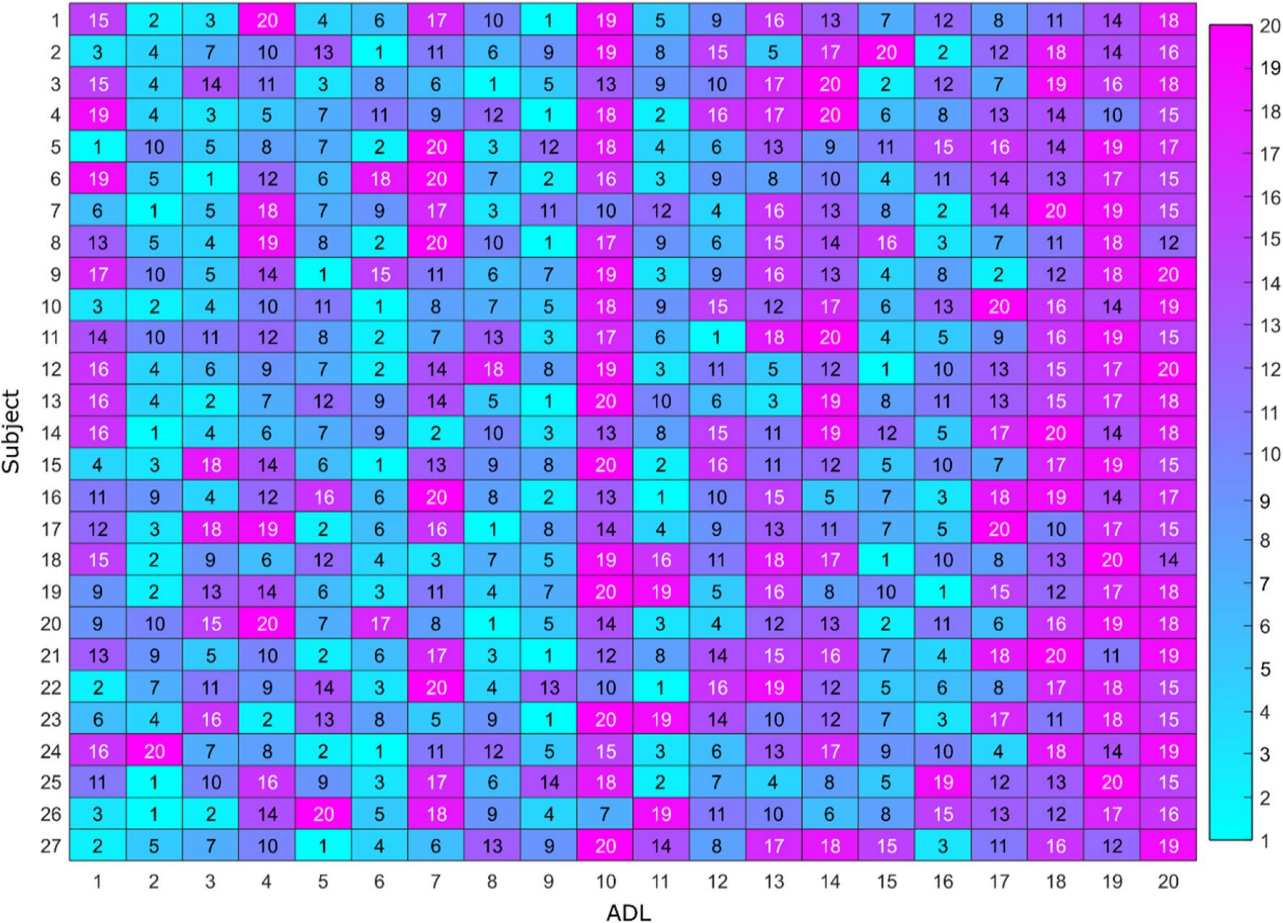
Cells contain the order of removal of ADLs (columns) for each healthy subject (rows) during the iterative process. Cells are coloured to represent the order of removal. Blue corresponds to 1 (the first removed ADL). Purple colour is represented by 20, and indicates the last removed ADL

Figure 4 shows the matrix of ADLs (columns) that contains, for each subject (rows), the order of the removal of ADLs. Some patterns appeared between subjects. For example, task #1 was removed in the first nine iterations for 74% of subjects, task #2 was removed in the first nine iterations for 50% of subjects, or task #4, which was removed in the first nine iterations for 62% of subjects. Similarly, activities #10, #13, #14, #17, #18, #19 and #20 were removed in the 10 last steps for at least 50% of subjects.

### Step 3—ADL selected per subject

**Fig. 5:**
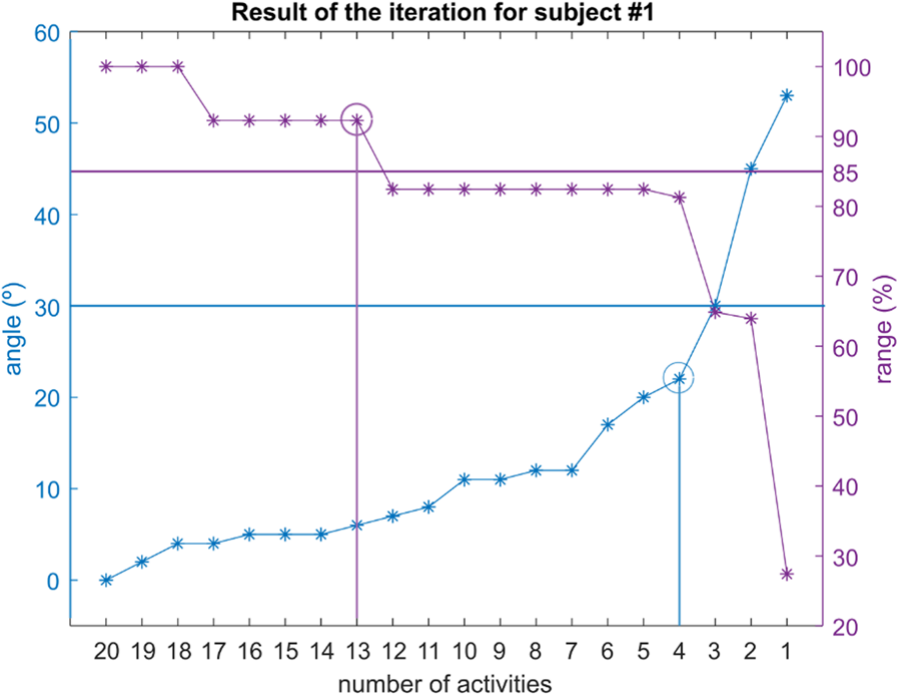
Example of the result of the iterative method per subject #1

To better understand the results obtained from the iterative process, the iteration result from subject #1 is illustrated in Fig. 5 as an example of the method for one subject. The X-axis shows the number of activities considered in each k-step. The left Y-axis indicates the maximum level of similarity between the Ref PC and the k-PCs. The right Y-axis displays the range of scores (%) between the k-PCs and the corresponding Ref PCs.

In this case, according to the first criterion (blue horizontal line), four ADLs would be required to keep the angle equal or lower than 30° (blue vertical line). According to the second criterion (purple horizontal line), 13 ADLs would be needed to maintain the range of the scores equal or higher than 85% (purple vertical line). Therefore, in this case, the stricter criterion was the second one and 13 ADLs were selected.

**Fig. 6:**
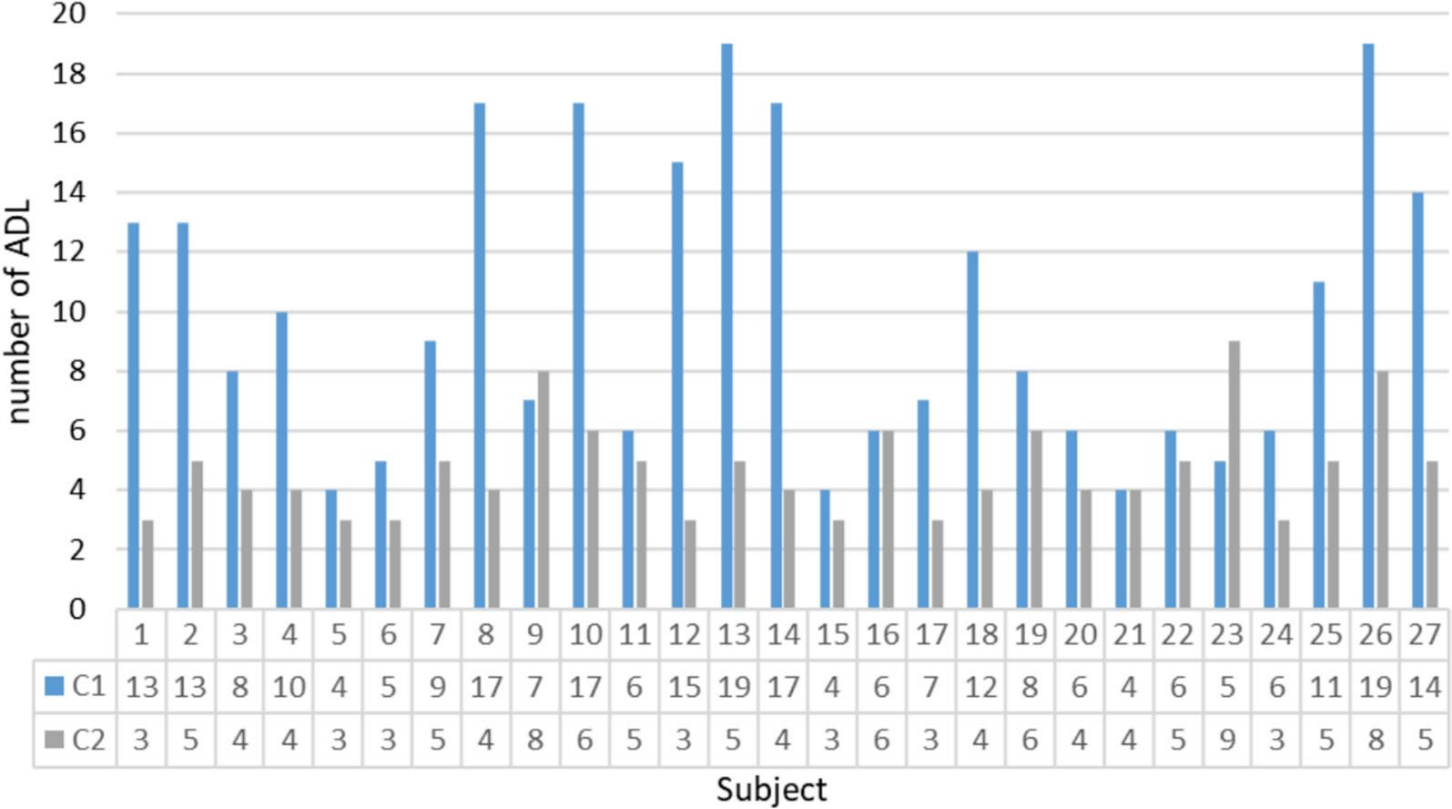
Number of ADLs required per subject to meet each criterion. C1 corresponds to the range criterion (angle equal or lower than 30°). C2 corresponds to the similarity criterion (the range of the scores equal or higher than 85%)

Figure 6 shows the results of the iterative method for all 27 subjects, where the number of ADLs required per subject to accomplish each criterion is observed. In general, the range criterion was more restrictive than the angle similarity one. Only subjects #9 and #23 required more ADLs (8 and 9 ADLs, respectively) to keep angles lower than 30° than to maintain the range above 85%.

**Fig. 7:**
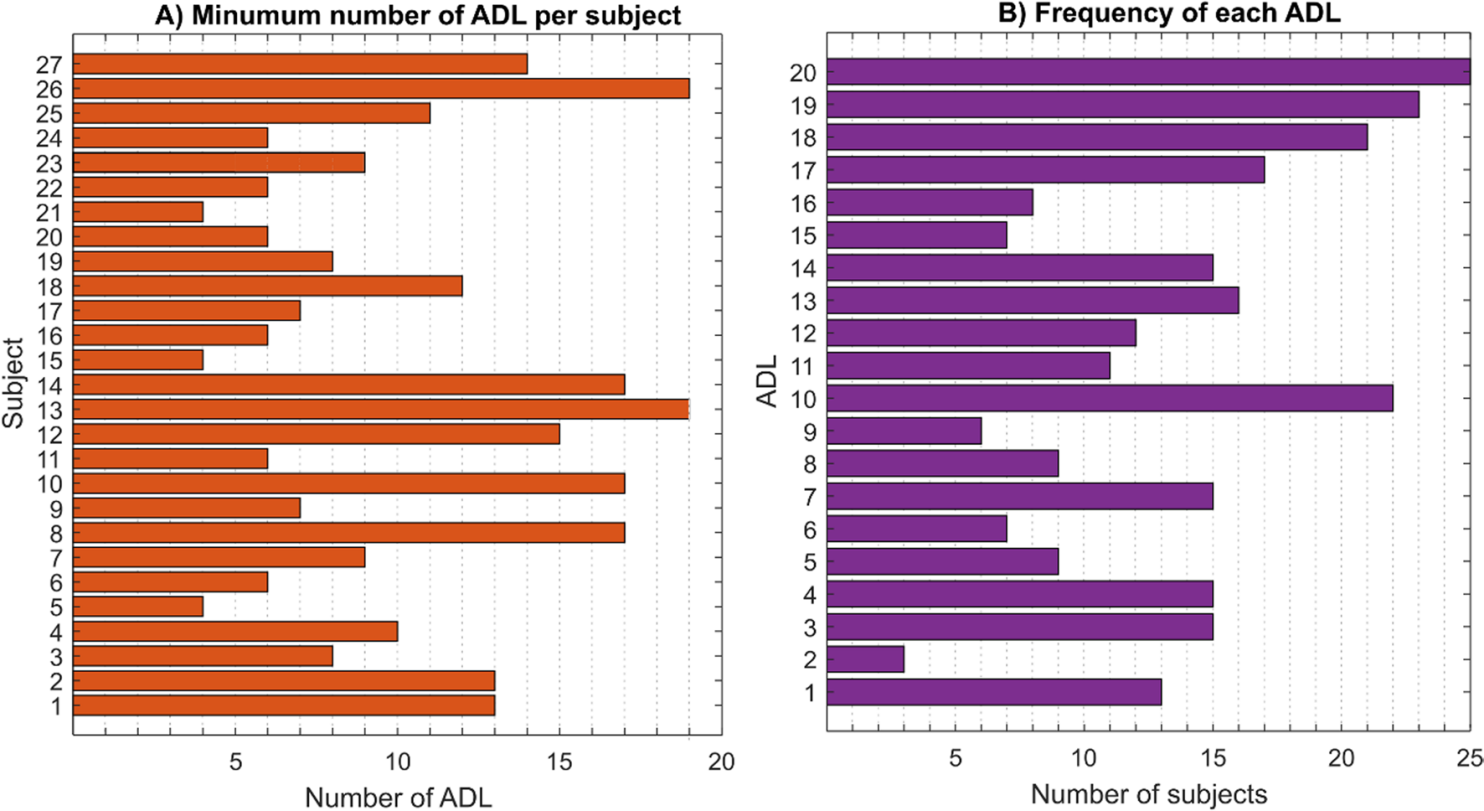
**A** Minimum number of ADLs required per subject; **B** frequency of each ADL obtained from the minimum set of ADLs per subject

Figure 7A shows the minimum number of ADLs required for all the 27 subjects according to the most restrictive criterion. Subjects #13 and #26 required almost all the ADLs, while subjects #5, #15 and #21 only required four ADLs. The average number of ADLs was 9.9 with a standard deviation (SD) of 4.9.

Figure 7B shows the frequency of each ADL occurring in the minimum set of ADLs per subject. Activities #10, #19 and #20 were the most frequent and appeared in more than 21 subjects. Activity #2 was the least frequent and appeared in only three subjects. The average number of frequencies of ADLs was 13.45 subjects with a standard deviation of 6.1. Ten ADLs were, therefore, the most frequent ones, and were finally selected for the BE-UJI set. They are detailed in next step.

### Step 4—reduced set of ADLs for the hand function assessment

Table 2 shows the grasp used in each ADL, the frequency (indicated as the number of subjects who exhibited specific activity among their iterative process) and the description of the reduced set of 10 selected ADLs following the aforementioned criteria.

**Table 2:** Description of the ADLs selected for the smaller set of ADLs

Grasp	Description	ADL	Frequency
Pulp pinch	Pour water from a cup	20	25
Transvers volar grip	Pour water from a jar	19	23
Five-finger pinch	Unscrew lid of jars	10	22
Pulp pinch	Pour water from Pure-Pak	18	21
Diagonal volar grip	Lift telephone receiver, put to ear	17	17
Diagonal volar grip	Cut play-doh with a knife and fork	13	16
Tripod pinch	Write with pen	14	15
Lateral pinch	Pick up nuts and turn them until completely screwed onto bolts	7	15
Five-finger pinch	Lift wooden cubes over edge 5 cm in height	4	15
Lateral pinch	Pick up coins from purses	3	15

### Step 5—task effect on the main kinematic parameters

#### Healthy cohort

For all the 27 subjects from the healthy cohort, Table 3 shows the angle between the Ref PCs and the BE-UJI reduced set PCs and the average angle (µ) across PCs. The averaged µ across the subject was 20.35° with an SD of 8.88°. Subject #20 presented the worst level of similarity (38.8°), and Ref PC4 was the PC with the least similarity (63°). Subject #17 presented the best similarity level (8.5°) and Ref PC2 was the PC with the most similarity (13°).

**Table 3:** Angle and average angle (µ) in degrees between the Ref PCs and the Set PCs for the healthy cohort

Subject	Ref PC1	Ref PC2	Ref PC3	Ref PC4	µ
1	10	18	28	26	20.5
2	6	28	28	58	30.0
3	9	8	13	18	12.0
4	20	32	22	22	24.0
5	7	15	8	11	10.3
6	6	10	15	9	10.0
7	14	19	14	25	18.0
8	17	5	29	19	17.5
9	35	38	44	26	35.8
10	6	25	18	12	15.3
11	6	12	7	17	10.5
12	28	13	29	51	30.3
13	27	33	19	32	27.8
14	12	14	15	14	13.8
15	37	32	18	48	33.8
16	9	9	11	30	14.8
17	6	13	5	10	8.5
18	8	7	22	12	12.3
19	6	10	16	31	15.8
20	24	29	39	63	38.8
21	5	9	30	45	22.3
22	31	28	30	21	27.5
23	11	24	32	55	30.5
24	14	43	27	24	27.0
25	8	12	31	12	15.8
26	7	6	10	37	15.0
27	5	12	11	21	12.3

#### HOA patients

Similarly to Table 3, for all the 33 HOA patients, Table 4 shows per subject the angle between the Ref PCs and the set PCs and the average angle (µ) across PCs. The averaged µ across the subject was 25.5° with an SD of 5.96°. Subject #14 presented the worst level of similarity (35.8°), with Ref PC3 being the PC with the least similarity (55°). Subject #22 presented the best level of similarity (11.8°), with Ref PC1 being the PC with the most similarity (7°).

**Table 4:** Angle and the average angle (µ) in degrees between the Ref PCs and the set PCs for HOA patients

Subject	Ref PC1	Ref PC2	Ref PC3	Ref PC4	µ
1	26	28	25	36	28.8
2	9	15	28	52	26
3	12	24	17	33	21.5
4	19	22	39	21	25.3
5	13	27	35	38	28.3
6	15	25	46	44	32.5
7	26	5	37	35	25.8
8	23	44	22	40	32.3
9	20	17	34	51	30.5
10	20	38	17	21	24
11	31	14	40	29	28.5
12	14	23	34	22	23.3
13	4	16	33	61	28.5
14	34	27	55	27	35.8
15	33	28	36	25	30.5
16	18	13	37	17	21.3
17	40	18	33	26	29.3
18	16	17	42	53	32
19	40	28	18	22	27
20	16	27	36	35	28.5
21	8	14	22	20	16
22	7	10	18	12	11.8
23	13	24	19	12	17
24	22	37	25	33	29.3
25	18	18	20	71	31.8
26	8	17	30	23	19.5
27	13	9	20	22	16
28	9	9	47	30	23.8
29	35	23	37	30	31.3
30	8	13	8	21	12.5
31	19	14	29	41	25.8
32	10	34	14	35	23.3
33	14	13	29	42	24.5

Additional file 1: Figs. S1–S6 show the boxplots of the range of motion and velocities of hand joints across subjects (healthy cohort: Additional file 1: Figs. S1–S3; HOA patients: Additional file 1: Figs. S4–S6) by considering all the SHFT ADLs (all the frames of all the ADLs) and only the reduced set (all the frames of the selected ADLs). In general, the range of motion of all the joints of both populations was equal or higher in the reduced set of ADLs. In particular, thumb IP joint and MCP joint of fingers presented the biggest difference between both sets of ADLs. For velocities, values were generally similar between sets of activities. In the healthy cohort, PIP joints of index and middle fingers presented the greatest decrease (flexion of PIP2 from 135°/s to 120°/s; extension of PIP2 from 130°/s to 120°/s). Similarly for HOA patients, PIP joint of index finger presented the greatest decrease (flexion of PIP2, from 120°/s to 110°/s; extension of PIP2, from 120°/s to 110°/s).

## Discussion

Herein a set of 10 tasks, the BE-UJI activity set, was found to be the equivalent to the full set of SHFT tasks in terms of hand kinematics requirements. The SHFT tasks were originally intended to be representative of hand function in day-to-day life by considering the commonest handgrips used while performing ADLs. Therefore, the BE-UJI activity set can be used for hand function assessments by monitoring hand kinematics requirements when performing ADLs to provide objective information about motion strategies, ranges of motion and velocities. The main advantage of this activity set is its shorter test duration, which could potentially lead to a 50% reduction in the time required for the SHFT. This reduction in testing time can be particularly beneficial because it addresses a current obstacle in the clinical evaluation of individuals with pathologies.

The reduction of tasks was performed efficiently by comparing subject-specific hand kinematics in terms of synergies (first four synergies), instead of comparing the original 16 joint angles. The kinematic synergies herein obtained per subject after considering all the SHFT tasks were similar to those reported in the literature [20, 27, 29, 31]: the first two synergies were related to fingers flexion and adduction, and were similar among subjects. The third and fourth synergies showed independence of thumb [28, 32], and were more diverse between subjects.

During the ADLs ordering process, many activities followed similar patterns between subjects, and some activities were removed in the last part of the iteration. This could allow the results herein found to be extrapolated to the global population to, therefore, reduce the total number of ADLs required to monitor hand kinematics. Particularly from these ordered ADLs, we selected those ADLs required to ensure not only certain bounded kinematics similarity (< 30°), but also functional joint ranges (> 85%). The range criterion was generally more restrictive than similarity, i.e. keeping hand joint coordination required fewer ADLs than keeping range of motion. It should be noted that some subjects would have enough with four tasks, while others would require almost all the ADLs. These differences between subjects could be due to each subject’s specific properties, such as the morphology of the hand itself, or even their previous experience when performing ADLs. However, the task activity set herein defined aims to be representative of not only a specific subject, but also of all the subjects on the whole. Thus the tasks that appeared more frequently were selected (Table 2), which reduced tasks to half from 20 to only 10. The obtained set included varied tasks that ranged from those using a grasp that was maintained throughout the task, like pouring water, to those that required more manipulation, like picking up coins from purses. Table 2 also shows the main grasps used in the BE-UJI activity set tasks according to Sollerman [17].

We found that the percentage of using the seven commonest handgrips in ADLs would be ensured with the 10 obtained tasks: 2 pulp pinch, 2 lateral pinch and 2 five-finger pinch; 1 tripod pinch; 2 diagonal volar grip and 1 transvers volar grip. Note that the removed ADLs required mostly pulp pinch or lateral pinch. Hence the proposed synergy-based methodology would also ensure the original ADLs function representativeness in terms of grasps.

The proposed synergy-based methodology guarantees kinematics in terms of ranges of motion, velocities and strategies. The strategies found in each set of activities were extremely similar [µ(SD): 20(9) degrees for the healthy cohort, 25.5(5.96) degrees for HOA patients]. Note that these angles were between PCs/synergies. Likewise, the ranges of motion obtained in both sets were extremely similar and of the same order of magnitude as the functional range of motion values that resulted from a previous work, which obtained a functional range of motion by considering real tasks [33]. The thumb IP and MCP joints were those with maximum differences (10°) and, in most cases, with wider ranges of motion in the BE-UJI set than in the 20 ADLs dataset. This was due to the way the range of motion was computed from percentiles P5 and P95. Eliminating the activities that contributed less to widen ranges of hand kinematics meant that percentiles moved more towards extremes. This also meant that P5 and P95 were higher than when considering all 20 activities. Velocities were also checked and were similar in the reduced test to those obtained with the 20 ADLs. PIP joints were those with the largest median differences and a slight drop of 15°/s.

The proposed reduced set of activities has been tested on an orthopaedic disability, such as HOA, and is based only on its representativeness in terms of kinematics. Further work should focus on studying its applicability to other pathologies, such as neurological diseases, where synergies may differ to a greater extent because of changes in neural coupling. The representativeness of the activity set in terms of force requirements should also be studied to validate the selection of tasks and/or to extend them if necessary.

## Conclusions

The provided BE-UJI activity set for hand function assessments can be used in clinics as an alternative to the SHFT. It reduces the test time and allows clinicians to obtain the objective kinematic data of the motor strategies, ranges of motion and joint velocities used by patients.

### Supplementary Information

**Additional file 1: Figure S1.** Ranges of motion of joints considering all the SHFT test ADLS and the Be-uji set of ADLS. **Figure S2.** Ranges of joint velocities in the positive direction (flexion and abduction) considering all the SHFT test ADLS and the Be-uji set of ADLS. **Figure S3.** Ranges of joint velocities in the negative direction (extension and adduction) considering all the SHFT test ADLS and the Be-uji set of ADLS. **Figure S4.** Range of motion of joints considering all the SHFT test ADLS and the be-uji set of ADLS on HOA patients. **Figure S5.** Range of joint velocities in the positive direction (flexion and abduction) considering all the SHFT test ADLS and the be-uji set of ADLS on HOA patients. **Figure S6.** Range of joint velocities in the negative direction (extension and adduction) considering all the SHFT test ADLS and the be-uji set of ADLS on HOA patients.

## Data Availability

Not applicable.

## Abbreviations

ADLs: Activities of daily living
CMC: Carpometacarpal
DoF: Degrees of freedom
PCA: Principal component analysis
PCs: Principal components
IP: Interphalangeal
MCP: Metacarpophalangeal
PIP: Proximal interphalangeal
SD: Standard deviation
SHFT: Sollerman Hand Function Test
