# Supplementary material for: The BE-UJI hand function activity set: a reduced set of activities for the evaluation of the healthy and pathological hand
Source: J Neuroeng Rehabil. 2023 Sep 21;20:122. doi: 10.1186/s12984-023-01245-1 (PMC10514972; doi:10.1186/s12984-023-01245-1)

Additional file 1

Néstor J. Jarque-Bou, Verónica Gracia-Ibáñez, Margarita Vergara, Joaquín L. Sancho-Bru

July 26, 2023

1. Overview

This document provides the supplementary material to support the manuscript entitled "BE-UJI hand function activity set: a reduced set of activities for the evaluation of the healthy and pathological hand" submitted to Journal of NeuroEngineering and Rehabilitation.

2. Table of contents

S1. RANGES OF MOTION OF JOINTS CONSIDERING ALL THE SHFT TEST ADLS AND THE BE-UJI SET OF ADLS. **2**

S2. RANGES OF JOINT VELOCITIES IN THE POSITIVE DIRECTION (FLEXION AND ABDUCTION) CONSIDERING ALL THE SHFT TEST ADLS AND THE BE-UJI SET OF ADLS **3**

S3. RANGES OF JOINT VELOCITIES IN THE NEGATIVE DIRECTION (EXTENSION AND ADDUCTION) CONSIDERING ALL THE SHFT TEST ADLS AND THE BE-UJI SET OF ADLS **4**

S4. RANGE OF MOTION OF JOINTS CONSIDERING ALL THE SHFT TEST ADLS AND THE BE-UJI SET OF ADLS ON HOA PATIENTS. **5**

S5. RANGE OF JOINT VELOCITIES IN THE POSITIVE DIRECTION (FLEXION AND ABDUCTION) CONSIDERING ALL THE SHFT TEST ADLS AND THE BE-UJI SET OF ADLS ON HOA PATIENTS. . **6**

S6. RANGE OF JOINT VELOCITIES IN THE NEGATIVE DIRECTION (EXTENSION AND ADDUCTION) CONSIDERING ALL THE SHFT TEST ADLS AND THE BE-UJI SET OF ADLS ON HOA PATIENTS… **7**

S1. Ranges of motion of joints considering all the SHFT test ADLs and the BE-UJI set of ADLs. The red horizontal central mark in boxes is the median; the edges of boxes are the 25th and 75th percentiles; whiskers extend to 1.5-fold the interquartile range. Nomenclature: _F for flexion, _A for abduction; 1 to 5, digits. Joints: IP for interphalangeal joint, PIP for proximal interphalangeal joints, MCP for metacarpophalangeal joints, CMC for carpometacarpal joints, PalmArch for palmar arch

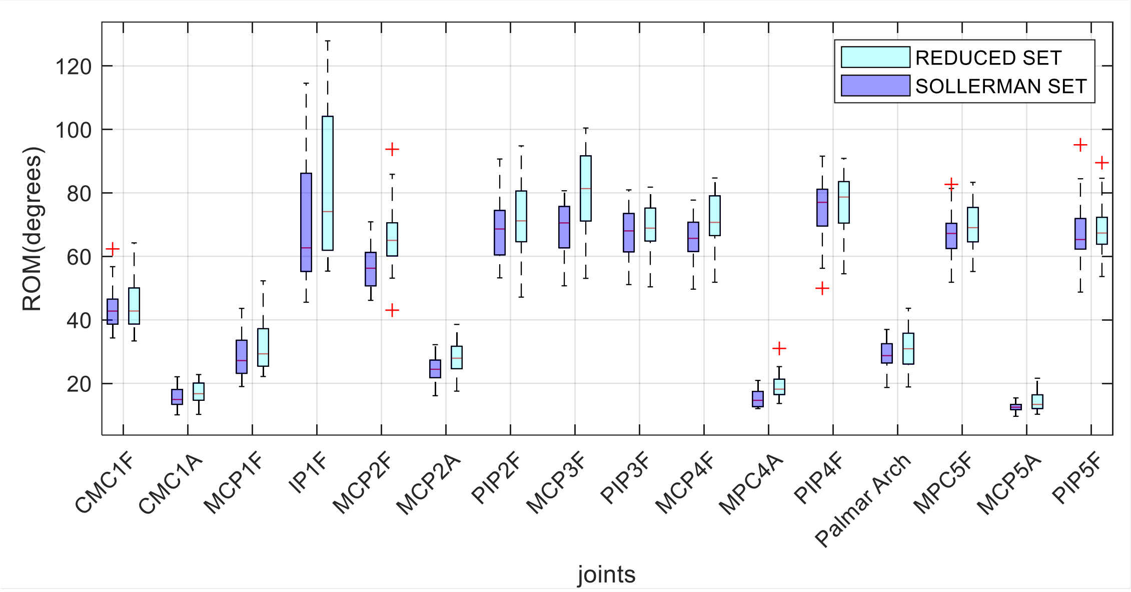

S2. Ranges of joint velocities in the positive direction (flexion and abduction) considering all the SHFT test ADLs and the BE-UJI set of ADLs. The red horizontal central mark in boxes is the median; the edges of boxes are the 25th and 75th percentiles; whiskers extend to 1.5-fold the interquartile range. Nomenclature: _F for flexion, _A for abduction; 1 to 5, digits. Joints: IP for interphalangeal joint, PIP for proximal interphalangeal joints, MCP for metacarpophalangeal joints, CMC for carpometacarpal joints, PalmArch for palmar arch

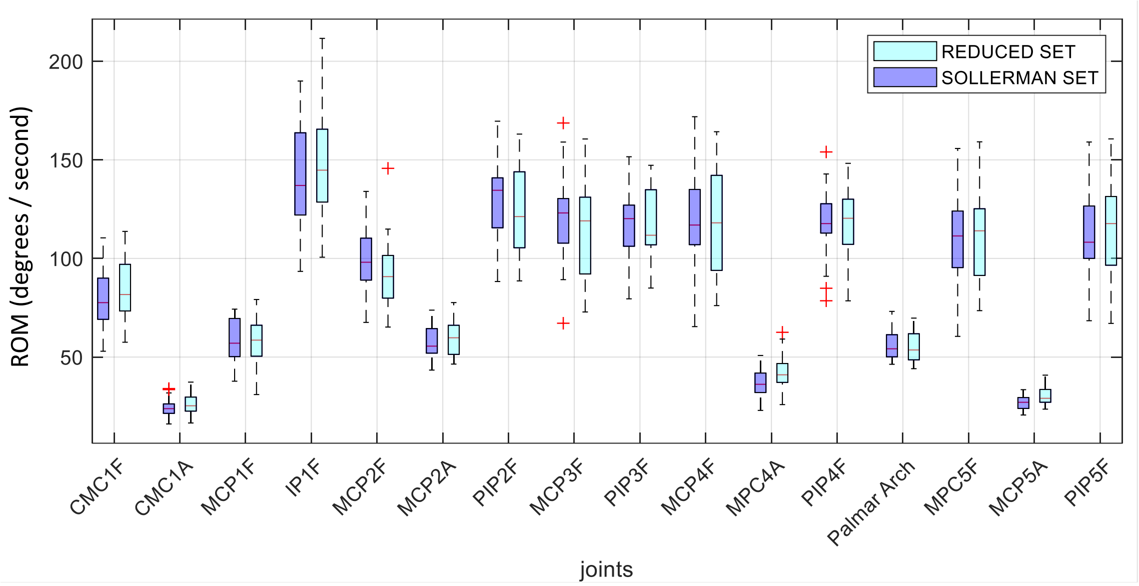

S3. Ranges of joint velocities in the negative direction (extension and adduction) considering all the SHFT test ADLs and the BE-UJI set of ADLs. The red horizontal central mark in boxes is the median; the edges of boxes are the 25th and 75th percentiles; whiskers extend to 1.5-fold the interquartile range. Nomenclature: _F for flexion, _A for abduction; 1 to 5, digits. Joints: IP for interphalangeal joint, PIP for proximal interphalangeal joints, MCP for metacarpophalangeal joints, CMC for carpometacarpal joints, PalmArch for palmar arch

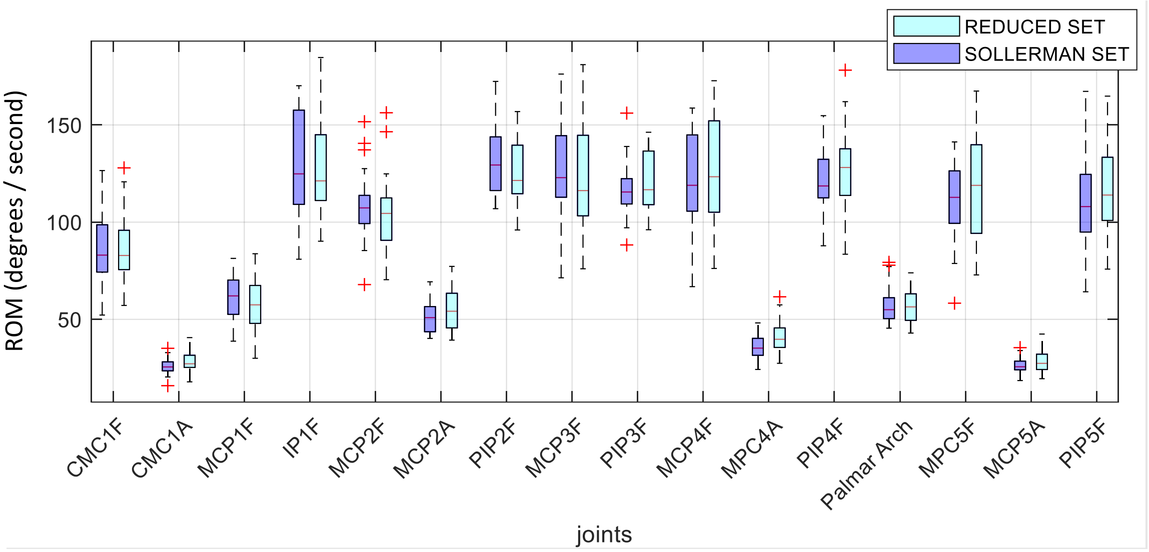

S4. Range of motion of joints considering all the SHFT test ADLs and the BE-UJI set of ADLs on HOA patients. The red horizontal central mark in the boxes is the median; the edges of the boxes are the 25th and 75th percentiles; whiskers extend to 1.5 times the interquartile range. Nomenclature: _F for flexion, _A for abduction; 1 to 5, digits. Joints: IP for interphalangeal joint, PIP for proximal interphalangeal joints, MCP for metacarpophalangeal joints, CMC for carpometacarpal joints, PalmArch for palmar arch

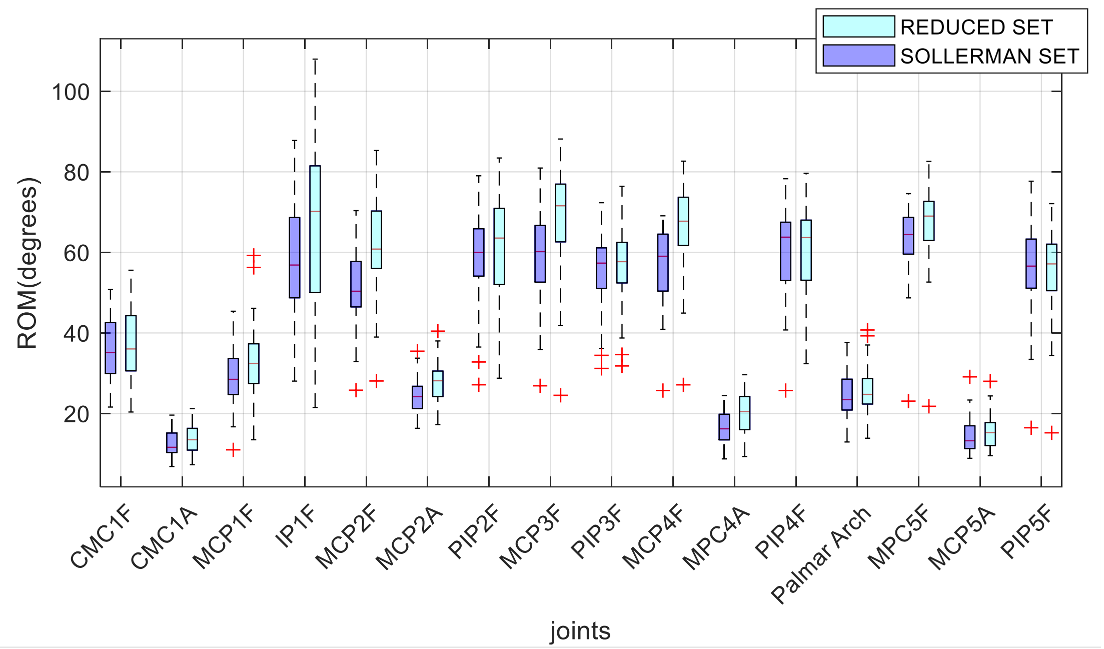

S5. Range of joint velocities in the positive direction (flexion and abduction) considering all the SHFT test ADLs and the BE-UJI set of ADLs on HOA patients. The red horizontal central mark in the boxes is the median; the edges of the boxes are the 25th and 75th percentiles; whiskers extend to 1.5 times the interquartile range. Nomenclature: _F for flexion, _A for abduction; 1 to 5, digits. Joints: IP for interphalangeal joint, PIP for proximal interphalangeal joints, MCP for metacarpophalangeal joints, CMC for carpometacarpal joints, PalmArch for palmar arch

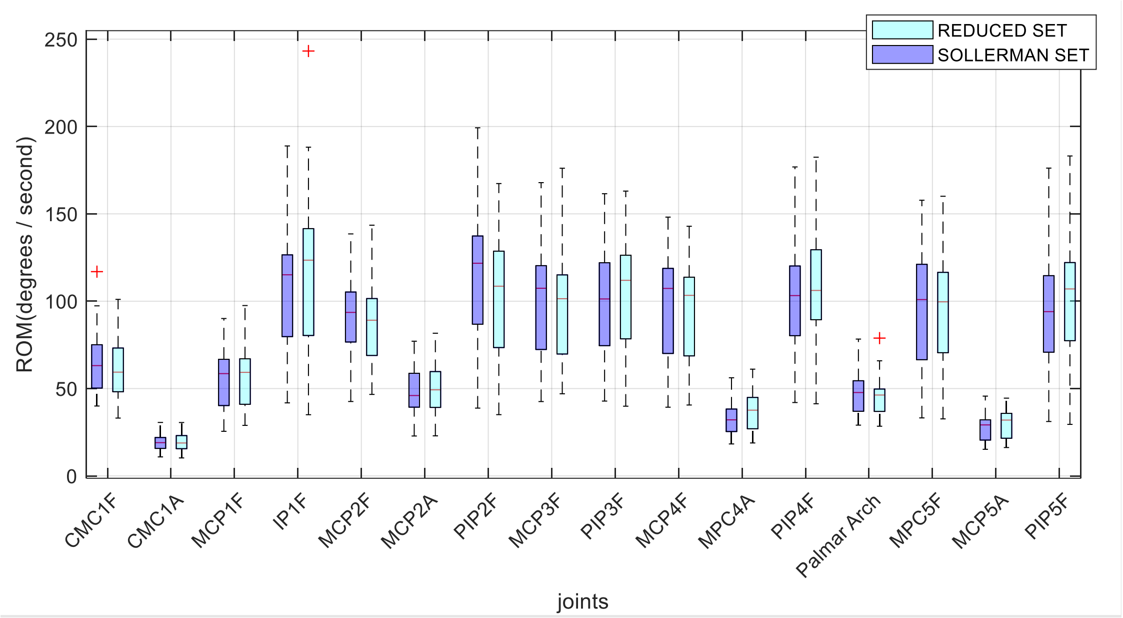

S6. Range of joint velocities in the negative direction (extension and adduction) considering all the SHFT test ADLs and the BE-UJI set of ADLs on HOA patients. The red horizontal central mark in the boxes is the median; the edges of the boxes are the 25th and 75th percentiles; whiskers extend to 1.5 times the interquartile range. Nomenclature: _F for flexion, _A for abduction; 1 to 5, digits. Joints: IP for interphalangeal joint, PIP for proximal interphalangeal joints, MCP for metacarpophalangeal joints, CMC for carpometacarpal joints, PalmArch for palmar arch

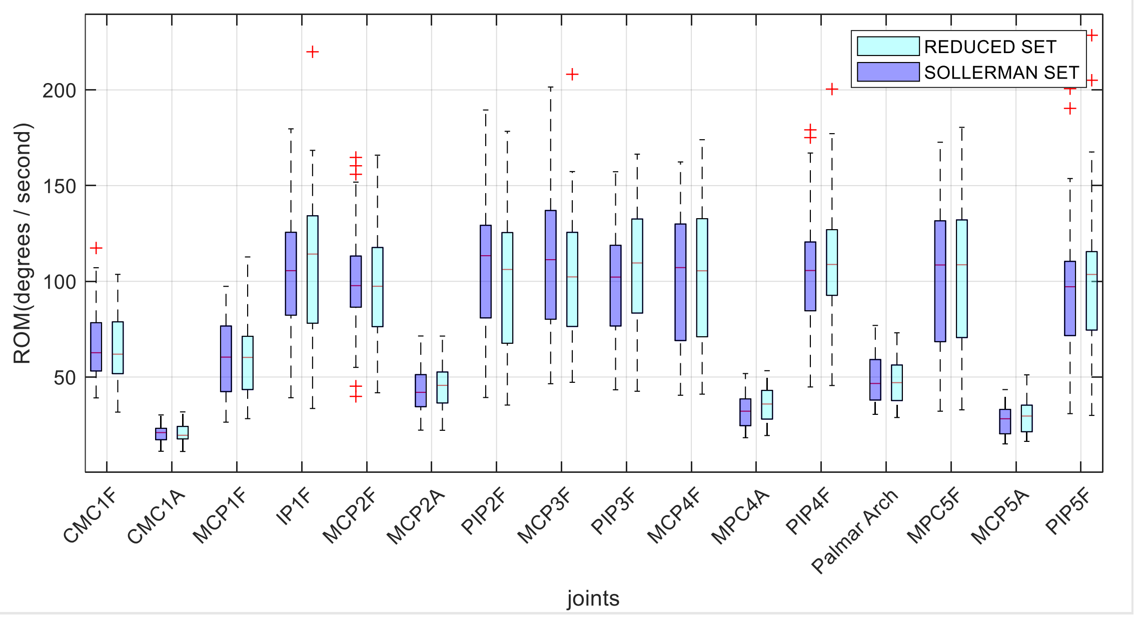

Supplement: Supplementary file 1 — Additional file 1: Figure S1. Ranges of motion of joints considering all the SHFT test ADLS and the Be-uji set of ADLS. Figure S2. Ranges of joint velocities in the positive direction (flexion and abduction) considering all the SHFT test ADLS and the Be-uji set of ADLS. Figure S3. Ranges of joint velocities in the negative direction (extension and adduction) considering all the SHFT test ADLS and the Be-uji set of ADLS. Figure S4. Range of motion of joints considering all the SHFT test ADLS and the be-uji set of ADLS on HOA patients. Figure S5. Range of joint velocities in the positive direction (flexion and abduction) considering all the SHFT test ADLS and the be-uji set of ADLS on HOA patients. Figure S6. Range of joint velocities in the negative direction (extension and adduction) considering all the SHFT test ADLS and the be-uji set of ADLS on HOA patients. [file 12984_2023_1245_MOESM1_ESM.pdf]
